# Supplementary material for: Geriatric ocular trauma and mortality: A retrospective cohort study
Source: PLoS One. 2025 May 28;20(5):e0324821. doi: 10.1371/journal.pone.0324821 (PMC12118826; doi:10.1371/journal.pone.0324821)
Supplement: Attachment — Submitted filename: Response to Additional Comments.docx [file pone.0324821.s003.docx]

1. In the online submission form you indicate that your data is not available for proprietary reasons and have provided a contact point for accessing this data. Please note that your current contact point is a co-author on this manuscript. According to our Data Policy, the contact point must not be an author on the manuscript and must be an institutional contact, ideally not an individual. Please revise your data statement to a non-author institutional point of contact, such as a data access or ethics committee, and send this to us via return email. Please also include contact information for the third party organization, and please include the full citation of where the data can be found.

Response: We provided many contact points and one of them was a co-author. We have removed the co-author as a contact point for accessing data but have kept the other contact points. For clarity, we have added the contact points below as well. We have updated the data availability statement removing our co-author as a contact point and have revised the data statement to a non-author institutional point of contact. We have included contact information for the third-party organization.

Sandy Barnhart: Social Research Specialist, e-mail: [sandy_barnhart@med.unc.edu](mailto:sandy_barnhart@med.unc.edu)

I2B2 and NCTracs Support Specialist: Joe Mosnier, [mosnier@unc.edu](mailto:mosnier@unc.edu) and nctracs@unc.edu

2. We note that there is identifying data in the Supporting Information file <Supplementary Files.xlsx>. Due to the inclusion of these potentially identifying data, we have removed this file from your file inventory. Prior to sharing human research participant data, authors should consult with an ethics committee to ensure data are shared in accordance with participant consent and all applicable local laws.

Response: Thank you for removing the supporting information files titled “supplementary files”.


Please remove or anonymize all personal information (<specific identifying information in file to be removed>), ensure that the data shared are in accordance with participant consent, and re-upload a fully anonymized data set. Please note that spreadsheet columns with personal information must be removed and not hidden as all hidden columns will appear in the published file.

Response: Data are in accordance with participant consent and any remotely potentially identifying information in our files have been removed to ensure full anonymity.
